# Chitosan Oligosaccharides Attenuate Amyloid Formation of hIAPP and Protect Pancreatic β-Cells from Cytotoxicity

**DOI:** 10.3390/molecules25061314

**Published:** 2020-03-13

**Authors:** Qin-Yu Meng, Hua Wang, Zi-Bo Cui, Wen-Gong Yu, Xin-Zhi Lu

**Affiliations:** Key Laboratory of Glycoscience & Glycotechnology of Shandong Province, School of Medicine and Pharmacy, Ocean University of China, Qingdao 266003, China; mengbinfang@126.com (Q.-Y.M.); fields17@hotmail.com (H.W.); cuizibo93@163.com (Z.-B.C.)

**Keywords:** chitosan oligosaccharides, type 2 diabetes, human islet amyloid polypeptide, amyloid, cytotoxicity

## Abstract

The deposition of aggregated human islet amyloid polypeptide (hIAPP) in the pancreas, that has been associated with β-cell dysfunction, is one of the common pathological features of patients with type 2 diabetes (T2D). Therefore, hIAPP aggregation inhibitors hold a promising therapeutic schedule for T2D. Chitosan oligosaccharides (COS) have been reported to exhibit a potential antidiabetic effect, but the function of COS on hIAPP amyloid formation remains elusive. Here, we show that COS inhibited the aggregation of hIAPP and disassembled preformed hIAPP fibrils in a dose-dependent manner by thioflavin T fluorescence assay, circular dichroism spectroscopy, and transmission electron microscope. Furthermore, COS protected mouse β-cells from cytotoxicity of amyloidogenic hIAPP, as well as apoptosis and cycle arrest. There was no direct binding of COS and hIAPP, as revealed by surface plasmon resonance analysis. In addition, both chitin-oligosaccharide and the acetylated monosaccharide of COS and glucosamine had no inhibition effect on hIAPP amyloid formation. It is presumed that, mechanistically, COS regulate hIAPP amyloid formation relating to the positive charge and degree of polymerization. These findings highlight the potential role of COS as inhibitors of hIAPP amyloid formation and provide a new insight into the mechanism of COS against diabetes.

## 1. Introduction

Type 2 diabetes (T2D), also known as non-insulin-dependent diabetes, is a widespread chronic disease characterized by insulin resistance, progressive loss of pancreatic β-cell function and mass, impaired insulin release, and hyperglycemia [1,2]. T2D is also an age-related disease prevalent in adults over 40 years old, and accounts for 90–95% of the total number of diabetic patients [3]. A variety of factors, including glycolipid toxicity, inflammation, and cholesterol accumulation have been reported to correlate with β-cell dysfunction and occurrence of T2D [4]. It is also suggested that the accumulation of aggregated islet amyloid polypeptides in the islets of Langerhans plays a critical role in pancreatic damage. 

Human islet amyloid polypeptide (hIAPP), alternate name amylin, is co-secreted with insulin by β-cells in the pancreas. Mature hIAPP contains 37 amino acids and is one of the most aggregation-prone peptides. It has been reported that aggregated hIAPP formed amyloid deposits in 70–90% of patients with T2D [5]. The amyloidogenic process of hIAPP for diabetes is shown in two aspects. First, small assemblies (usually called oligomers) of hIAPP exhibit a direct cytotoxicity on the β-cells [6]. Second, invasive amyloid deposits cause a strong inverse correlation with β-cell area [7,8]. Thus, it is necessary to develop potential inhibitors to prevent early aggregation of hIAPP and/or depolymerize its amyloid deposits in order to avoid irreparable damage to β-cells.

Chitosan is a product of partial deacetylation of chitin, which exists commonly in the exoskeletons of arthropods and insects and the cell walls of fungi. Chitosan oligosaccharides (COS), characterized as linear polymers of β-(1→4) linked d-glucosamine (GlcN) and *N*-acetyl-d-glucosamine (GlcNAc) residues with degree of polymerization (DP) less than 20 are derived from the hydrolysis of chitosan via physical, chemical, or enzymatic hydrolysis [9]. Compared with chitosan, COS have more bioavailability due to their lower molecular weight, higher solubility, and lower viscosity. Update, COS are the only positively charged oligosaccharides found in nature [10]. It has been demonstrated that COS possess diverse pharmacological activities and a broad range of applications [11]. Among them, the anti-diabetic bioactivity of COS has been extensively investigated by using various types of diabetic models [12]. COS were validated to be able to ameliorate glucose metabolism by improving glucose uptake, increasing insulin secretion, reducing insulin resistance, accelerating β-cell proliferation or neogenesis, and defending β-cells against apoptosis [13,14]. Mechanistically, COS suppress gluconeogenesis and stimulate glycogen synthesis in the liver through inhibition of p38 MAPK and phosphoenolpyruvate carboxykinase expression and AMPK activation with up-regulation of glucokinase expression [14]. In addition, COS may also improve glucose metabolism by reshaping the unbalanced gut microbiota of diabetic mice [15]. However, the direct biomolecules targeted by COS have not been revealed, and the correlation between COS and hIAPP amyloid formation remains unclear. Therefore, in this study, a battery of biophysical and cellular assays was performed to demonstrate the potential function of COS on hIAPP amyloid formation. Furthermore, we also preliminarily explored its underlying mechanism.

## 2. Results

### 2.1. Chitosan Oligosaccharides Preparation

**Figure 1:**
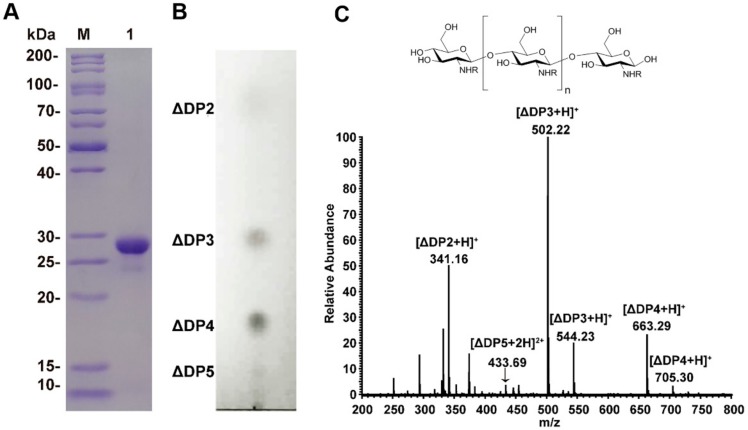
Analysis of purified CsnA and chitosan oligosaccharides. (**A**) SDS-PAGE analysis of CsnA. Lane M, protein molecular mass marker; Lane 1, purified CsnA. (**B**) TLC analysis of the hydrolysis product of CsnA. (**C**) The chemical structure and MS analysis of chitosan oligosaccharides. ‘R’ can be either H or acetyl group depending on the DP. DP2, (GlcN)_2_; DP3, (GlcN)_3_ and (GlcN)_2_(GlcNAc)_1_; DP4, (GlcN)_4_ and (GlcN)_3_(GlcNAc)_1_; DP5, (GlcN)_4_(GlcNAc)_1_; GlcN—2-amino-2-deoxy-d-glucose; GlcNAc—2-acetamido-2-deoxy-glucose; DP—degree of polymerization.

For COS preparation, we used recombinant chitosanase CsnA, an endo-type enzyme that has been shown to specially hydrolyze chitosan and generate chitobiose, chitotriose, and chitotetraose as main hydrolysates [16]. The purified enzyme had a purity over 90%, with a molecular weight of 28 kD on SDS-PAGE (Figure 1A). 

Enzyme hydrolysates were purified and enriched by ultrafiltration, nanofiltration, and rotary evaporation. Quality analysis showed that the final products contained 15.9 mg/mL of sugars with 25 μg/mL of residual proteins, and that endotoxin was under the detection limit. Further analysis of the sugar components using thin layer chromatography (TLC) and mass spectrometry (MS) methods indicated that the products were mainly tri-saccharides and tetra-saccharides, along with a small amount of di-saccharides and penta-saccharides (Figure 1B,C).

### 2.2. Fluorescent Assay of hIAPP Aggregation Influenced by COS 

**Figure 2:**
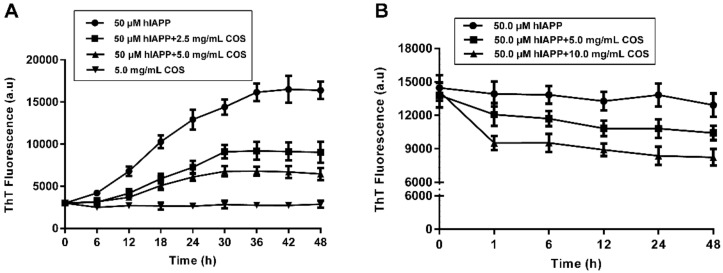
Thioflavin T (ThT) fluorescence analysis of chitosan oligosaccharides (COS) on human islet amyloid polypeptide (hIAPP) amyloid formation and disaggregation. (**A**) Effects of COS on hIAPP amyloid formation. (**B**) Effects of COS on the preformed hIAPP amyloid fibrils.

ThT is widely used as a fluorescence probe to track the amyloid deposits due to its fast, inexpensive, and reproducible fashion in the emission spectrum [17,18,19,20]. The effect of COS supplementation on the kinetics of hIAPP fibrillization was monitored over a period of 48 h. Samples were taken at time intervals to record ThT fluorescence. As shown in Figure 2A, the time-dependent kinetics of hIAPP aggregation were characterized by a typical S-shaped curve consisting of three phases: lag phase (formation of stable nuclei), elongation phase (elongation of nuclei to fibrils), and an equilibrium phase (floccule formation), similar with previous reported [21,22,23]. The fluorescence increase of hIAPP alone exhibits a short lag phase and a rapid growth phase up to 24 h, followed by reaching a plateau after further 12 h. Interestingly, COS drastically reduced fluorescence in a dose-dependent manner, confirming its inhibitory effect on hIAPP fibril formation. To rule out the interference of any residual protein, COS samples were boiled for ten minutes before use and similar results were observed (data not shown). 

The simplicity effect of COS on fibrillation dynamic was plotted as a function of time and fitted by a sigmoidal growth model [24]. It showed that both doses of COS nearly doubled the lag time of hIAPP aggregation, indicating the delaying effect of COS on hIAPP nucleation. Moreover, the fluorescence intensity values at the saturation phase decreased by nearly 46% for 2.5 mg/mL of COS and 60% for 5.0 mg/mL of COS, respectively (Table 1). The apparent aggregation constant *k* slightly increased with the presence of COS, suggesting a faster growth of hIAPP fibers after nucleation, which may reduce the formation of toxic intermediates. 

Disaggregating pre-existing hIAPP fibrils was an alternative treatment strategy for amyloid clearance. As shown in Figure 2B, the burst reduction of fluorescence occurred within the first hour, then tended to retardation. After 48 h of treatment with COS, the fluorescence intensities of hIAPP fibrils reduced by 11% for 5.0 mg/mL and 35% for 10.0 mg/mL, respectively.

The above results clearly demonstrated the role of COS in preventing the development of hIAPP monomers into fibrillary amyloid and disaggregating the preformed fibrils.

### 2.3. Secondary Structure Analysis of hIAPP Influenced by COS 

**Figure 3:**
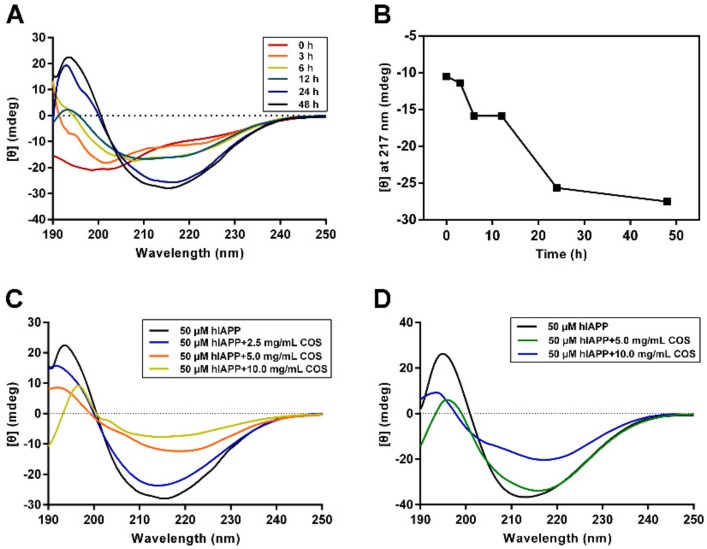
CD analysis of secondary structure transition of hIAPP. (**A**) Structural changes of hIAPP during fibrillation monitored by Far-UV CD. (**B**) Structural changes monitored at 217 nm. (**C**) COS affect hIAPP structure transition during aggregation. COS were used at the concentrations of 0, 2.5, 5.0 and 10.0 mg/mL. (**D**) COS disassemble of hIAPP fibrils at concentrations of 5.0 and 10.0 mg/mL.

Far-UV Circular Dichroism (CD) spectroscopy was used to provide a direct insight into the secondary structure transition of hIAPP during fibrillization [25]. Figure 3A showed that the CD spectra of hIAPP alone experienced a typical structural transition from random coil to β-sheet, as indicated by the appearance and intensity enhancement of the positive peak at 195 nm and the negative peak at 217 nm (Figure 3B). These two peaks correspond to the β-sheet structure, a characteristic feature for amyloid fibrils [26,27].

Three doses of COS (2.5, 5.0 or 10.0 mg/mL), based on the ThT result, were used to evaluate the influence on hIAPP conformational transition. The data recorded at 48 h indicated that COS significantly blocked the structural transition of hIAPP to β-sheet rich structure (Figure 3C). The characteristic peak intensity of the β-sheet at 195 nm decreased in a COS-concentration dependent manner. Of note, the COS-co-incubated hIAPP showed a similar trend of structural alteration with hIAPP alone, indicating that the function of COS was in preventing structural change, instead of forming new structures. Additionally, we conducted CD experiments on the disaggregation of preformed hIAPP fibrils by COS. The monitored second structure change of preformed hIAPP fibrils suggested that COS could partially disassemble the mature fibrils (Figure 3D). Consistent with the aggregation process, no new structural features were observed in the disaggregation process. 

### 2.4. Morphologies of hIAPP Aggregates Visualized by Transmission Electron Microscope 

**Figure 4:**
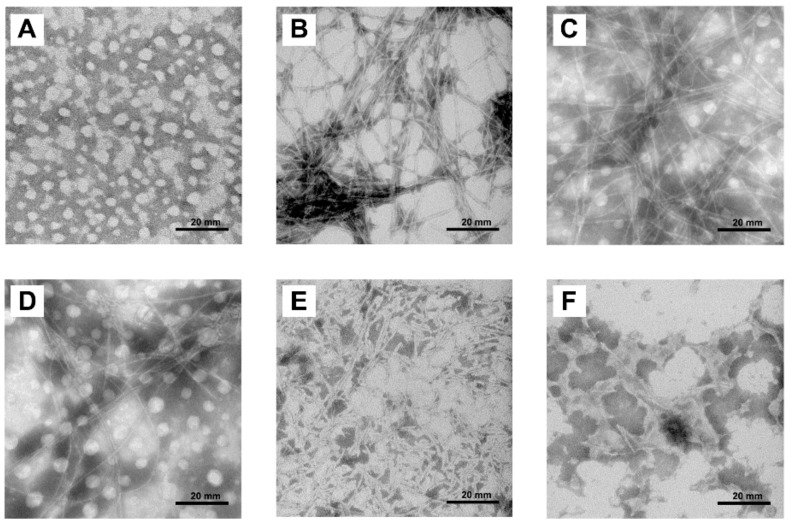
Morphology changes of hIAPP aggregates. Representative TEM images of (**A**) monomeric hIAPP. (**B**) formed fibrils by monomeric hIAPP after 48 h. (**C**) monomeric hIAPP co-incubated with 2.5 mg/mL of COS for 48 h. (**D**) monomeric hIAPP co-incubated with 5.0 mg/mL of COS for 48 h. (**E**) 5.0 mg/mL of COS on preformed hIAPP fibrils for 48 h. (**F**) 10.0 mg/mL of COS on preformed hIAPP fibrils for 48 h. Scale bar represents 20 mm.

The effect of COS on morphology changes of hIAPP during fibril formation was determined by transmission electron microscope (TEM). As shown in Figure 4, hIAPP alone showed typical amyloid morphology transition during the incubation. Monomeric hIAPP did not show any visible fibrillar structure, whereas upon 48 h incubation, it formed long, thick, unbranched fibers and crossed into a highly dense network (Figure 4A,B). On the other hand, the final fibers derived from COS treated hIAPP were slender and crossed into sparse mesh (Figure 4C,D). 

The disassemble ability of COS on mature hIAPP fibrils is shown in Figure 4E,F. Treated with 5.0 mg/mL of COS, the long hIAPP fibers were disrupted and fragmented, resulting in obvious rupture of the mesh. In contrast, 10.0 mg/mL of COS fractured the fibers into small pieces. Collectively, these results show that COS hindered the amyloid formation and disrupted the preformed amyloid of hIAPP.

### 2.5. Mechanism Study of hIAPP Aggregation Influenced by COS 

**Figure 5:**
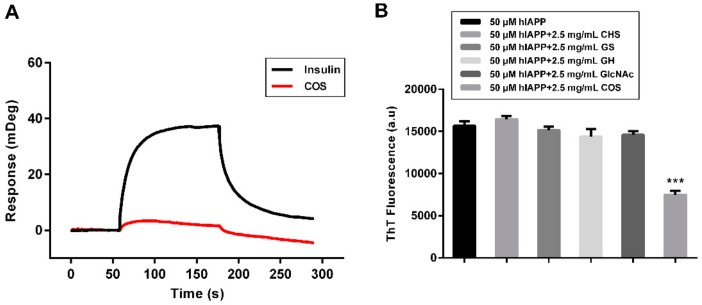
Binding affinity of COS with hIAPP and the effect of different oligosaccharides on hIAPP aggregation. (**A**) SPR detection of the binding between COS (red) and hIAPP. Mono-hIAPP was immobilized to the CM5 chip. Insulin (black) was used as the positive control. (**B**) ThT fluorescence detection of CHS (chitin oligosaccharides), GS (glucosamine sulfate), GH (glucosamine hydrochloride), GlcNAc (*N*-acetyl-d-glucosamine) on hIAPP amyloid fibrils formation.

To interpret the underlying mechanism of COS, we conducted surface plasmon resonance to directly evaluate the binding affinities between COS and hIAPP [28]. One of the main hydrolysis products, chitotetraose, was used for SPR analysis. The hIAPP peptide was coupled to the surface of the Biacore chip and increasing concentrations of chitotetraose were injected in a stepwise manner. Insulin was used for the positive control [29,30]. As shown in Figure 5A, no binding signal of COS to hIAPP was detected, suggesting that the function of COS on hIAPP aggregation was binding-independent.

Under physiological conditions, both COS and hIAPP are positively charged, underlying the electrostatic interactions between the two molecules. Therefore, we checked the effect of chitin oligosaccharides (CHS), with identical concentration and polymerization to experimental COS. CHS have similar constituents as COS, but are neutrally charged. As assessed by monitoring ThT fluorescence, CHS failed to prevent the fibrillization of hIAPP, emphasizing the importance of the positive charges (Figure 5B). Invalid effects on hIAPP aggregation were also shown in three monosaccharides, *N*-acetyl-d-glucosamine (GlcNAc), glucosamine sulfate (GS), and glucosamine hydrochloride (GH). These findings indicate that the function of COS not only relates to the charge, but the degree of polymerization.

### 2.6. Effect of COS on hIAPP Cytotoxicity 

**Figure 6:**
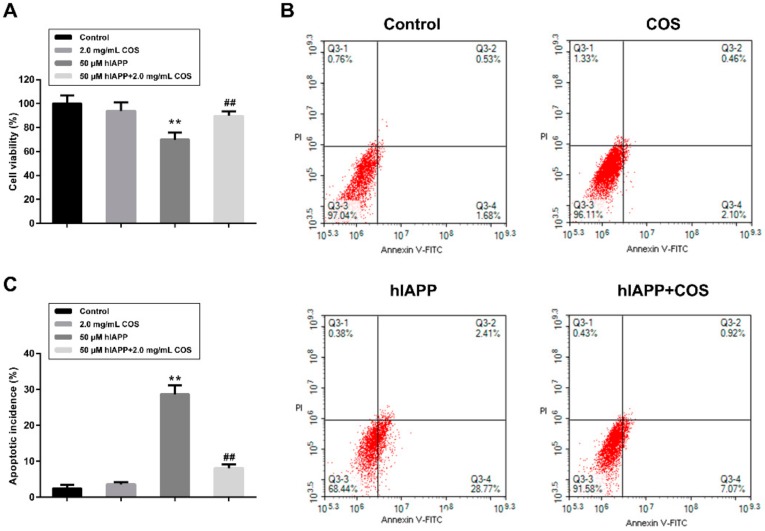
Effect of COS on hIAPP induced cytotoxicity to β-TC-6 cells. (**A**) Lactate dehydrogenase release analysis. Cells were treated with 50 μM hIAPP alone or co-treated with 2.0 mg/mL of COS for 24 h. (**B**) Representative graphs obtained by flow cytometry using double staining with Annexin V-FITC (a marker for apoptosis) and PI (a marker for necrosis). (**C**) The apoptotic incidence of β-TC-6 cells exposed to 50 μM hIAPP in the presence or absence of 2.0 mg/mL COS for 24 h. Data are expressed as means ± SD of three independent experiments. ** *p* < 0.01 vs. Control, ## *p* < 0.01 vs. hIAPP group.

Considering that amyloid formation may lead to the failure of islet β-cells, we conducted a cell viability experiment by lactate dehydrogenase (LDH) release and flow cytometry assay with β-TC-6 cells to examine the protective role of COS. LDH analysis was done to explore the integrity of the cell membrane in the presence of hIAPP amyloid. The concentration of hIAPP used was 50 μM, where obvious amyloid formation was observed during the incubation. The results showed that cell viability decreased to 75% after exposure to hIAPP for 24 h. At 2.0 mg/mL, COS alone showed no obvious LDH release induction, however, when incubated with the peptide together, COS significantly prevented hIAPP induced LDH release (Figure 6A).

Flow cytometry was performed to further preliminarily demonstrate hIAPP-induced apoptosis and necrosis in β-TC-6 cells. Annexin V vs. PI are presented in Figure 6B, which were the markers for apoptosis necrosis, respectively. As displayed in Figure 6B,C, cells treated with COS showed almost the same results as the negative control. On the contrary, a severe increase of apoptosis cells was observed (Q3-2 and Q3-4, 31.18%) after hIAPP treatment for 24 h. Also, it can be observed that COS effectively rescued pancreatic cells from apoptosis induced by hIAPP, confirming that COS could alleviate hIAPP-induced apoptosis.

**Figure 7:**
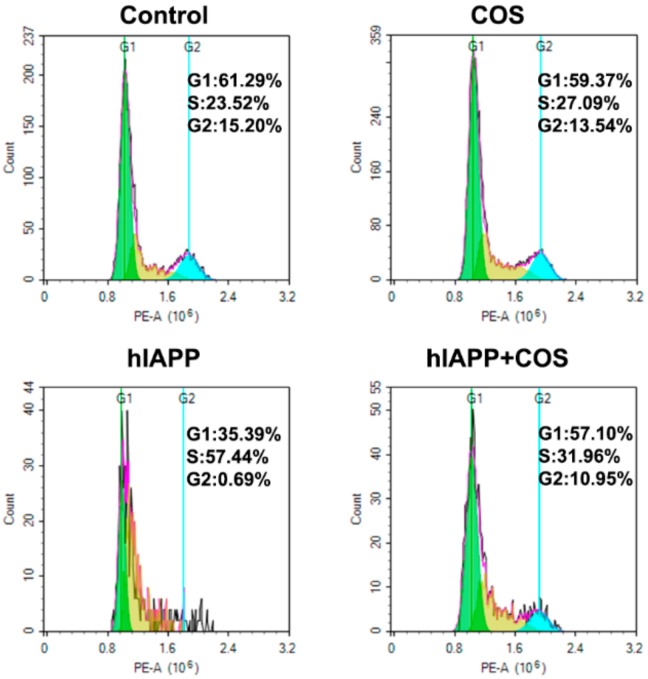
The effect of COS on hIAPP-induced cycle arrest in β-TC-6 cells. The G1, S and G2 phases in cell cycle are represented by green, yellow, and blue, respectively. The cells were exposed to 50 μM hIAPP in the presence or absence of 2.0 mg/mL COS for 24 h.

Furthermore, hIAPP treatment caused a remarkable cell cycle arrest at S phase, highlighting the β-cell proliferation inhibition effect of amyloidogenic hIAPP (Figure 7). This finding may explain, at least in part, the failure of correct expansion of β-cell mass. No remarkable change of cell cycle was observed when treated with COS. In contrast, COS relieved the cycle arrest induced by hIAPP. Based on the above results, it can be concluded that COS may ameliorate hIAPP-induced cytotoxicity, apoptosis, and cycle arrest of β-TC-6 cells.

## 3. Discussion

T2D is on the rise worldwide and the number of T2D patient is predicted to reach as many as 438 million by 2030 [31]. One of the hallmark features of T2D is the misfolding and aggregation of functional hIAPP into inactive amyloid fibrils, followed by deposition in the pancreatic islets, leading to cellular damage and dysfunction. Therefore, preventing and/or reversing the process of hIAPP aggregation provides an important therapeutic strategy for T2D.

A growing body of evidences indicate that COS can inhibit the aggregation of Aβ and reduce its neurotoxicity, exerting an anti-Alzheimer’s activity [25,32,33]. Considering that T2D has comparable pathophysiological features with Alzheimer’s disease, we propose that the antidiabetic function of COS owns to the mechanism of anti-amyloid formation of hIAPP. The effect of COS against hIAPP amyloid formation was verified by ThT, CD, and TEM methods. Moreover, COS not only inhibit hIAPP aggregation, but also disrupt the existing hIAPP fibrils in a concentration-dependent manner. In the aggregation stage, COS retard the nucleation process of hIAPP and decrease the amount of amyloid fibrils. However, the secondary structure transmission of hIAPP treated with COS, revealed by CD, was similar with that of hIAPP alone, suggesting that COS influenced hIAPP aggregation by inhibiting the binding between hIAPP molecules, rather than changing the secondary structure of hIAPP. Consistently, although slender and sparse, COS-treated hIAPP still aggregated into fiber-like networks. This mechanism was also reinforced by the affinity analysis, as no binding signals were observed between COS and hIAPP. On the other hand, our study proved that the inhibition effect of COS relates to its charge property. The positively charged property of COS was also correlated to its anticancer, anti-bacteria, and anti-obesity bioactivities [34,35]. Therefore, we propose that COS enhance the intermolecular electrostatic repulsion of hIAPP, accordingly reducing the aggregation and destroying the formed fibrils.

The cytotoxicity of amyloidogenic hIAPP has been widely reported [36,37,38]. Here we found aggregated hIAPP induced β-cell apoptosis and inhibited cell proliferation. These results may partially explain β-cell loss and dysfunction in T2D. Previous reports showed that COS act as antidiabetic agents by protecting pancreatic β-cells related to immunopotentiation and antioxidation [39]. In this study, we provide the direct evidence that COS protect β-cells by alleviating the cytotoxicity of amyloidogenic hIAPP. The mechanism of COS counteracting with hIAPP amyloid needs to be studied further.

Collectively, these findings provide a reasonable mechanistic link between anti-amyloid formation and the antidiabetic effects of COS. Given the availability, low toxicity, and high bio-tolerance, COS as a potential therapeutic agent for the treatment of T2D deserves further study.

## 4. Materials and Methods 

### 4.1. Materials 

Recombinant strain *E. coli* BL21(DE3)/pET24a(+)-csnA for expressing the chitosanase was previously constructed in our laboratory [16]. Human IAPP (> 95%) was synthesized by Gen Script (Nanjing, China). Thioflavin T (ThT) and 1,1,1,3,3,3-hexafluoro-2-propanol (HFIP) were purchased from Sigma-Aldrich (St. Louis, MO, USA). Annexin V-FITC apoptosis detection kit I was obtained from Vazyme Biotech (Najing, China). Chitosan (C90% deacetylated) was purchased from Qingdao MdBio, Inc. (Qingdao, China). The silica gel plates 60F 254 for thin layer chromatography (TLC) was purchased from Merck (Darmstadt, Germany).

### 4.2. Purification of Recombinant Enzymes 

The expression and purification of recombinant enzymes were conducted as our previous report [40]. Briefly, recombinant *E. coli* BL21(DE3)/pET24a(+) -csnA was induced with 0.5 mM IPTG (Beyotime Biotechnology, Shanghai, China) at 25 °C, 160 rpm for 60 h. The supernatants were harvested by centrifugation at 10000 × g for 20 min at 4 °C and further subjected to the Ni-Sepharose column. The purity and molecular weight of the protein were analyzed by SDS-PAGE. The protein concentration was detected using BCA protein assay kit (Beyotime Biotechnology, Shanghai, China). Enzyme activity was determined by 3,5-dinitrosalicylic acid (DNS) method.

### 4.3. Preparation of Chitosan Oligosaccharides 

For the preparation of chitosan oligosaccharides, the procedure was conducted as described previously [40]. Initially, 10 grams of water-soluble chitosan was dissolved in 1 L of water, and then 10 mL of purified CsnA (162 U/mL) was added. The mixture was incubated and stirred at 37 °C. The hydrolysis process was monitored at 30 min intervals until finished. A continuous hydrolysis process was performed by adding 1 g of substrate every 2 h to a reach the final concentration of 10%. The final hydrolysis supernatant was harvested by centrifugation at 10000 × g for 20 min at 4 °C and stored at 4 °C.

An ultrafiltration membrane with a molecular weight rejection of 8000 Da was used to remove macromolecular polysaccharides and proteins from hydrolysis solution. A nanofiltration filter with a 200 Da cut-off molecular weight was used to desalinate. The solution was concentrated by rotary evaporation and further freeze-dried. Finally, the products were stored at −20 °C before use.

The products were assayed by sulfuric acid-phenol method for sugar content, BCA method for protein content, and hydrazine reagent gel method for endotoxin content. The constituents of the oligosaccharides were analyzed using thin layer chromatography (TLC) and further identified by mass spectrometry (MS).

### 4.4. hIAPP Preparation and Aggregation 

Synthesized hIAPP was dissolved in HFIP at a final concentration of 1 mM to remove pre-existing aggregates, and the solvent was completely removed by freeze-drying in a vacuum freeze-dryer. The peptide was stored at −20 °C until use.

Before experiment, the lyophilized hIAPP was dissolved in 2 mM HCl, sonicated for 10 min, and centrifuged at 16000 × g for 20 min at 4 °C. The solution was diluted with 20 mM Tris-HCl buffer (pH 7.4) and incubated at 37 °C for aggregation assay.

The effect of COS on hIAPP aggregation was evaluated by dissolving COS in freshly prepared hIAPP monomer solutions to the final concentrations of 2.5 and 5.0 mg/mL. For the disassemble assay, hIAPP fibrils were prepared by incubating at 37 °C for 48 h and verified by ThT fluorescence assay to assure mature fibril formation. Then COS were added and incubated with hIAPP for another 48 h.

### 4.5. Thioflavine T (ThT) Fluorescence Assay 

ThT fluorescence assay was used to monitor the progress of hIAPP fibril formation and the preformed fibrils’ disruption in the presence or absence of COS. Samples were diluted 19-fold with ThT solution (10 μM) (Sigma-Aldrich, Saint Louis, MO, USA). The hIAPP aggregation status at designated points was determined by ThT fluorescence assay and recorded on a Multimode Plate Reader (PerkinElmer EnSpire, Waltham, MA, USA) at 482 nm with an excitation wavelength of 440 nm. The fluorescence intensity of solution without hIAPP was subtracted from that of solution containing hIAPP to deduct background fluorescence. Data were representative of three independent experiments.

### 4.6. Circular Dichroism Spectroscopy 

Far ultraviolet circular dichroism (CD) spectra of the hIAPP solutions were measured at a concentration of 0.2 mg/mL in 20 mM Tris-HCl buffer (pH 7.4), using a Jasco J-810 spectropolarimeter (Jasco Corp., Tokyo, Japan) with a 0.1 cm path-length quartz cuvette. Spectra were recorded in triplicate scans with a step size of 0.5 nm and a bandwidth of 1 nm; the ellipticity data were collected from 190 to 250 nm. A background value for each test was subtracted from the corresponding value of each sample, and the spectra were smoothed using the FFT filter function of the Jasco software. The curve of hIAPP coupled with COS was obtained by subtracting the background with same concentration of COS.

### 4.7. Transmission Electron Microscopy (TEM) 

TEM measurements were performed at different time intervals to characterize the morphological changes of hIAPP aggregates in the presence or absence of COS. Samples were negatively stained with 1.5% (wt./vol.) uranyl acetate solution on grids (400 mesh) covered by carbon-coated collodion film. The morphology of amyloid fibers was observed and photographed by a JEM-1200 EX transmission electron microscope (JEOL, Tokyo, Japan) operated at 100 kV after drying.

### 4.8. Cell Culture

Mouse insulinoma (β-TC-6) cells were obtained from the Chinese Academy of Science (Shanghai, China). The cells were cultured in Dulbecco’s modified Eagle’s medium (DMEM) supplemented with 15% fetal bovine serum (FBS) (Gibco, Grand Island, NY, USA), penicillin (100 U/mL) and streptomycin (100 μg/mL) at 37 °C with a 5% CO2 injection.

### 4.9. Cytotoxicity Assay and Cell Cycle Analysis

Lactate dehydrogenase release (LDH) and flow cytometry were employed to measure the toxicity of hIAPP. β-TC-6 cells were seeded in 96-well culture plate at a density of 1 × 10^4^/ well and incubated at 37 °C for 48 h. Then the cells were exposed to hIAPP (50 μM), COS (2.0 mg/mL), or the mixture of COS and hIAPP for 24 h. Cell viability was measured by LDH assay following the manufacture’s instruction (Beyotime Biotechnology, Shanghai, China). The experiment was performed in triplicate. For apoptosis analysis, the treated cells were incubated with Annexin V-FITC/PI for 20 min in the dark at room temperature, following the manufacturer’s instructions (Beyotime Biotechnology, Shanghai, China). Then, cells were analyzed by flow cytometry assay using NovoCyte D3080 and visualized by NovoExpress (ACEA, Los Angeles, CA, USA).

Cell cycle study was also performed with flow cytometry. Briefly, after treatment, β-cells were harvested and subsequently fixed in 70% (*v/v*) chilled ethanol overnight at 4 °C. Then the cells were washed with PBS and subsequently resuspended in PBS and incubated with PI and RNase (Beyotime Biotechnology, Shanghai, China) for 30 min at room temperature. Finally, the samples were applied to flow cytometry analysis. 

### 4.10. Surface Plasmon Resonance (SPR)

Purified chitotetraose was used to test the binding ability between COS and hIAPP on a Biacore T200 instrument (GE Healthcare, USA) in PBS buffer at 25 °C. The mono-hIAPP at a concentration of 100 μg/mL was immobilized on a CM5 sensor chip (GE Healthcare, USA) at a density of 400 response units. Chitotetraose (2.5 μM) was diluted in PBS buffer and passed over the CM5 sensor chip at a flow rate of 10 μL/min. Human insulin was used as the positive control [27,28]. The binding of analytes to the immobilized hIAPP resulted in a change of refractive index. The response was measured using SPR and compared with the control sample (an activated and blocked flow-cell without hIAPP) on the same chip. The experiments were repeated three times. 

### 4.11. Statistical Analysis

All experiments were performed in triplicate, and the data were expressed as means ± SD of three independent experiments. Statistical evaluation was performed by one-way analysis of variance (ANOVA), followed by post-hoc Student–Newman–Keulsmethods. A level of *p* < 0.05 was considered to be statistically significant.

## 5. Conclusions

In summary, hIAPP is a major component of amyloid deposits found in pancreatic β-cells of T2D. While COS not only reduced significantly the aggregation of hIAPP, they also disassembled preformed hIAPP fibrils in a dose-dependent manner. Furthermore, COS protected mouse β-cells from cytotoxicity of amyloidogenic hIAPP, as well as apoptosis and cycle arrest. COS regulating hIAPP amyloid formation may relate to their positive charge and degree of polymerization. Thus, COS may be considered as promising inhibitors of hIAPP aggregation to treat T2D. Moreover, in the later studies, we will make efforts to identify the active component from the COS mixture and give deep insight into its antidiabetic mechanism.

## Figures and Tables

**Table 1:** Kinetics of hIAPP, incubated in the absence or presence of COS.

Sample	t_1/2_ (h)^1^	Lag Time (h)^2^	*k* (h^−1^)^3^	Maximum Intensity (a.u.) ^4^
hIAPP	14.83 ± 0.96	4.30 ± 0.36	0.19 ± 0.01	16060 ± 206.40
hIAPP + COS-2.5 mg/mL	17.63 ± 0.23	9.00 ± 0.43	0.23 ± 0.012	8640 ± 226.50
hIAPP + COS-5.0 mg/mL	15.91 ± 0.76	8.47 ± 0.80	0.28 ± 0.03	6410 ± 124.90

^1^ t_1/2_ represents the aggregation time corresponding to 50% of the maximum fluorescence intensity.^2^ Lag time is defined as the time when the slope at the point of maximum fibrillation intersects the abscissa. ^3^ *k* belongs to the kinetic constant, which is defined as the apparent first-order aggregation constant. ^4^ Maximum intensity is the maximum fluorescence intensity.
